# Supplementary figures and images for: A chimeric nuclease substitutes a phage CRISPR-Cas system to provide sequence-specific immunity against subviral parasites
Source: eLife. 2021 Jul 7;10:e68339. doi: 10.7554/eLife.68339 (PMC8263062; doi:10.7554/eLife.68339)

Figure 4- source data 1

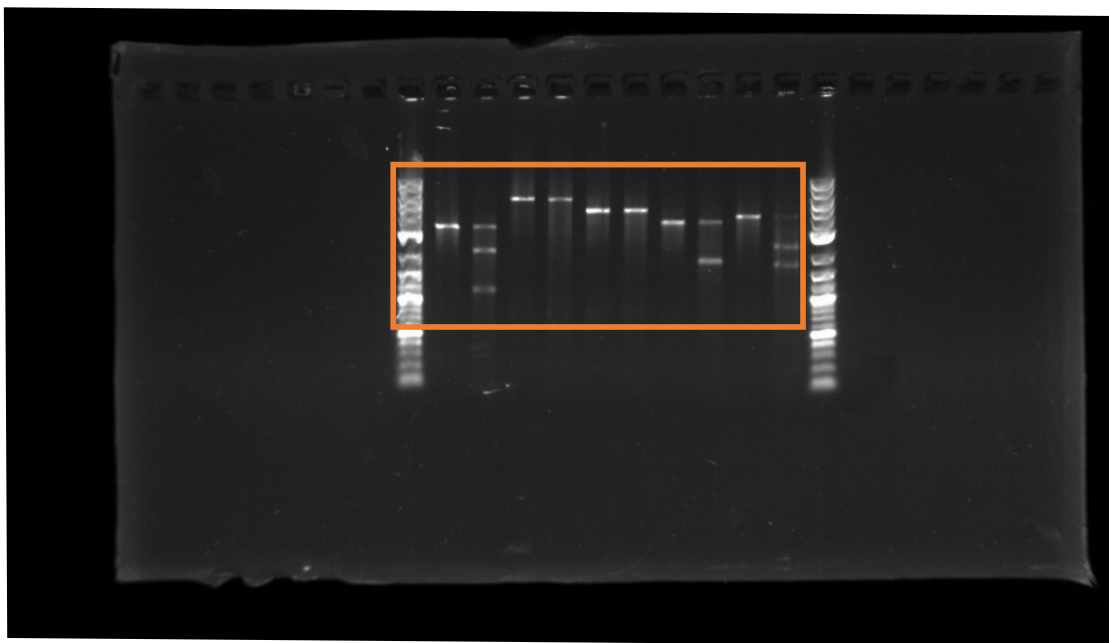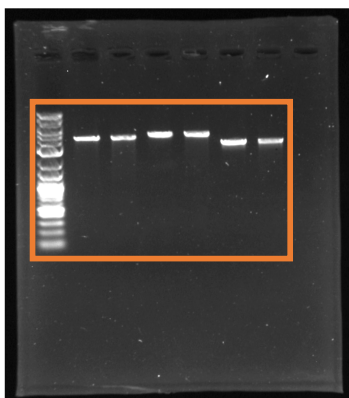

Supplement: Figure 4—source data 1. — The cropped images shown in the figure are indicated by the orange boxes. [file elife-68339-fig4-data1.pdf]

Figure 4- figure supplement 3- source data 1

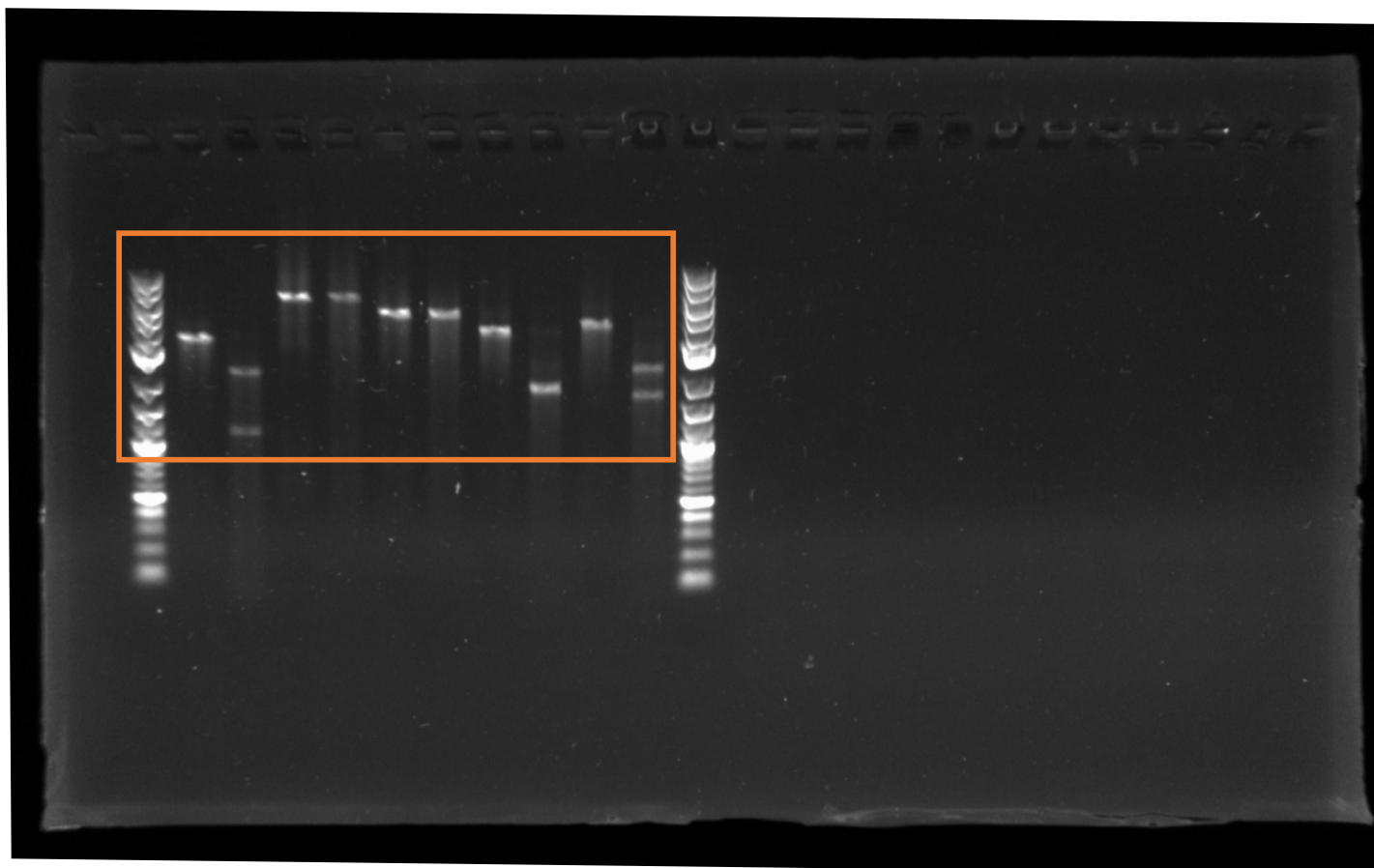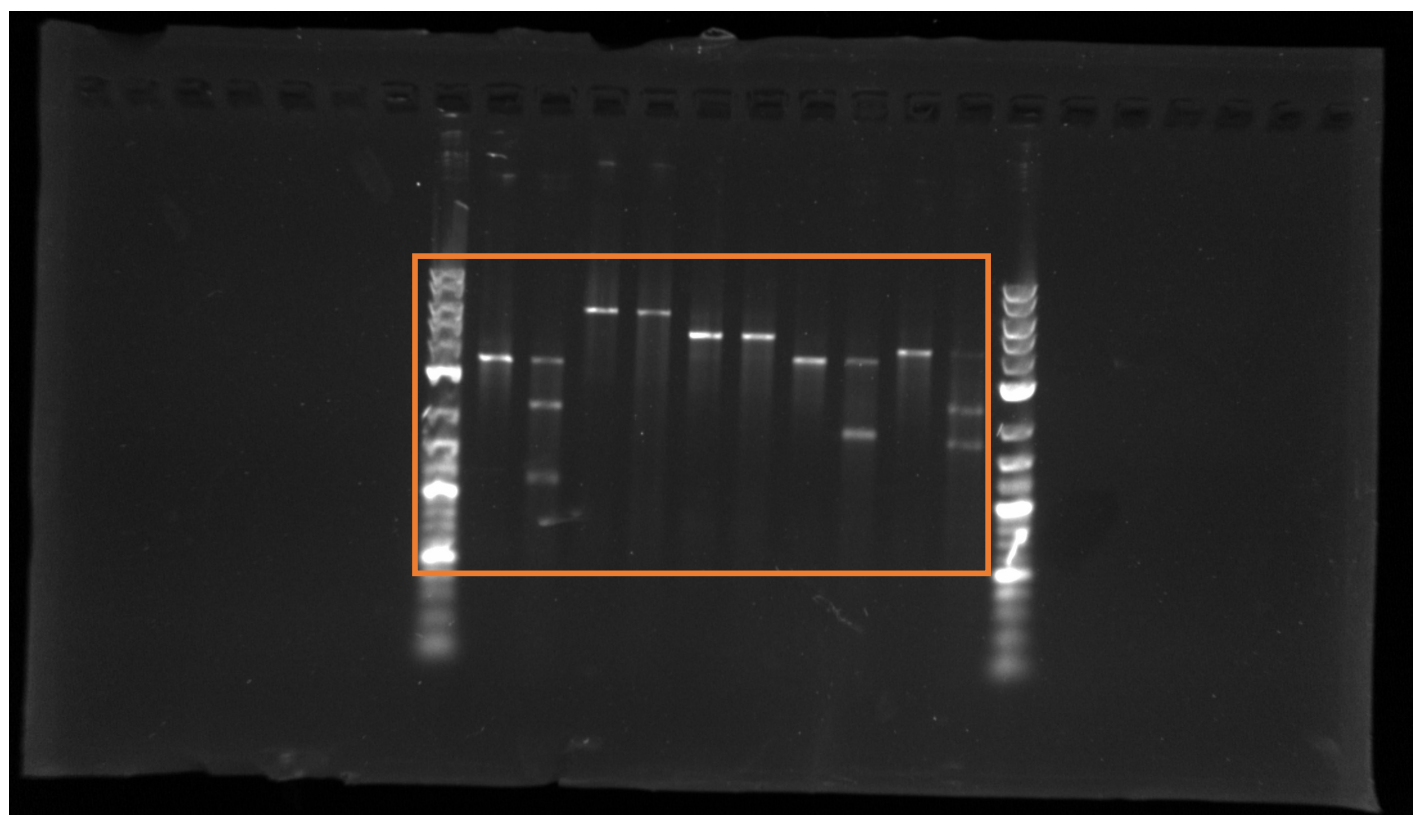

Supplement: Figure 4—figure supplement 3—source data 1. — The cropped images shown in the figure are indicated by the orange boxes. [file elife-68339-fig4-figsupp3-data1.pdf]

Figure 4- figure supplement 4- source data 1

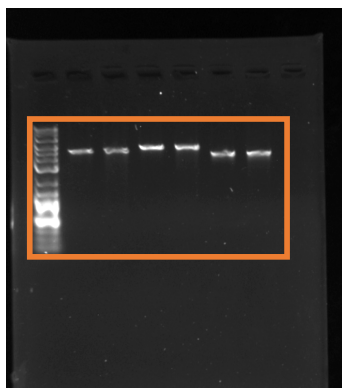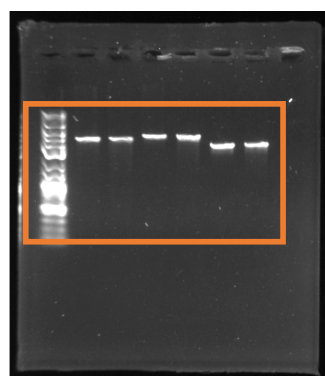

Supplement: Figure 4—figure supplement 4—source data 1. — The cropped images shown in the figure are indicated by the orange boxes. [file elife-68339-fig4-figsupp4-data1.pdf]

Figure 4- figure supplement 5- source data 1

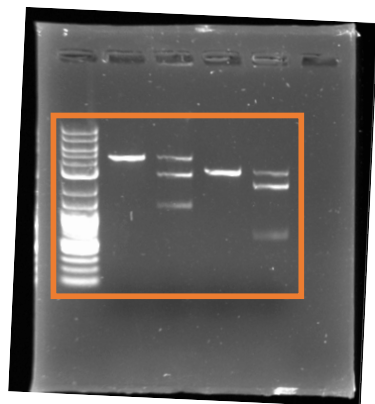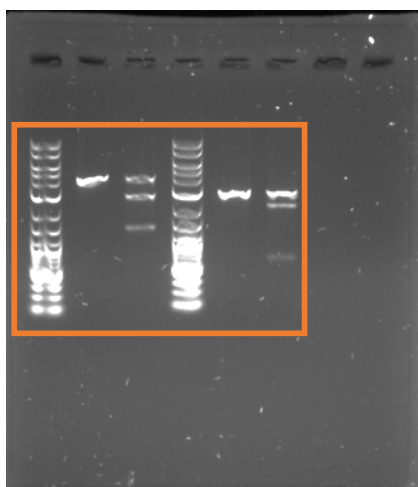

Supplement: Figure 4—figure supplement 5—source data 1. — The cropped images shown in the figure are indicated by the orange boxes. [file elife-68339-fig4-figsupp5-data1.pdf]

Figure 5-source data 1

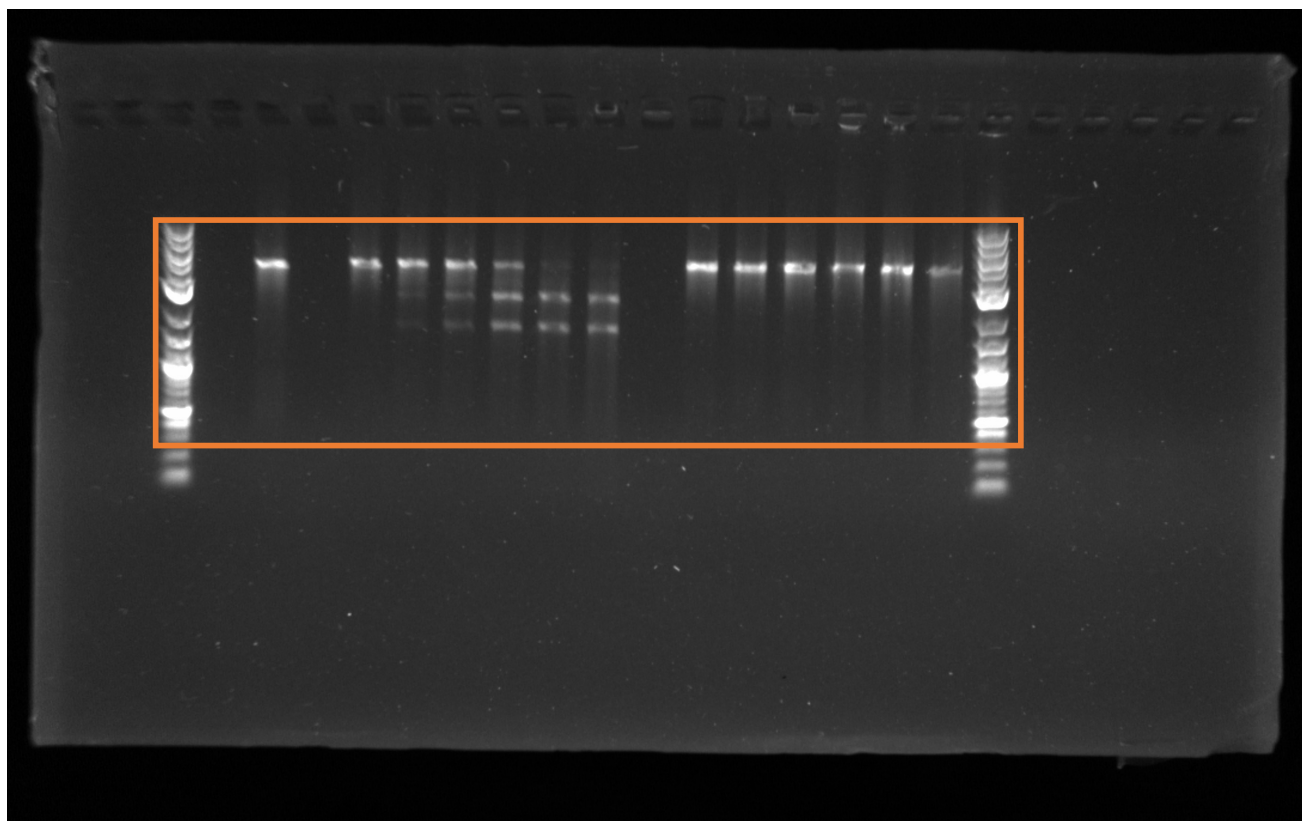

Supplement: Figure 5—source data 1. — The cropped image shown in the figure is indicated by the orange box. [file elife-68339-fig5-data1.pdf]

Figure 5- figure supplement 1- source data 1

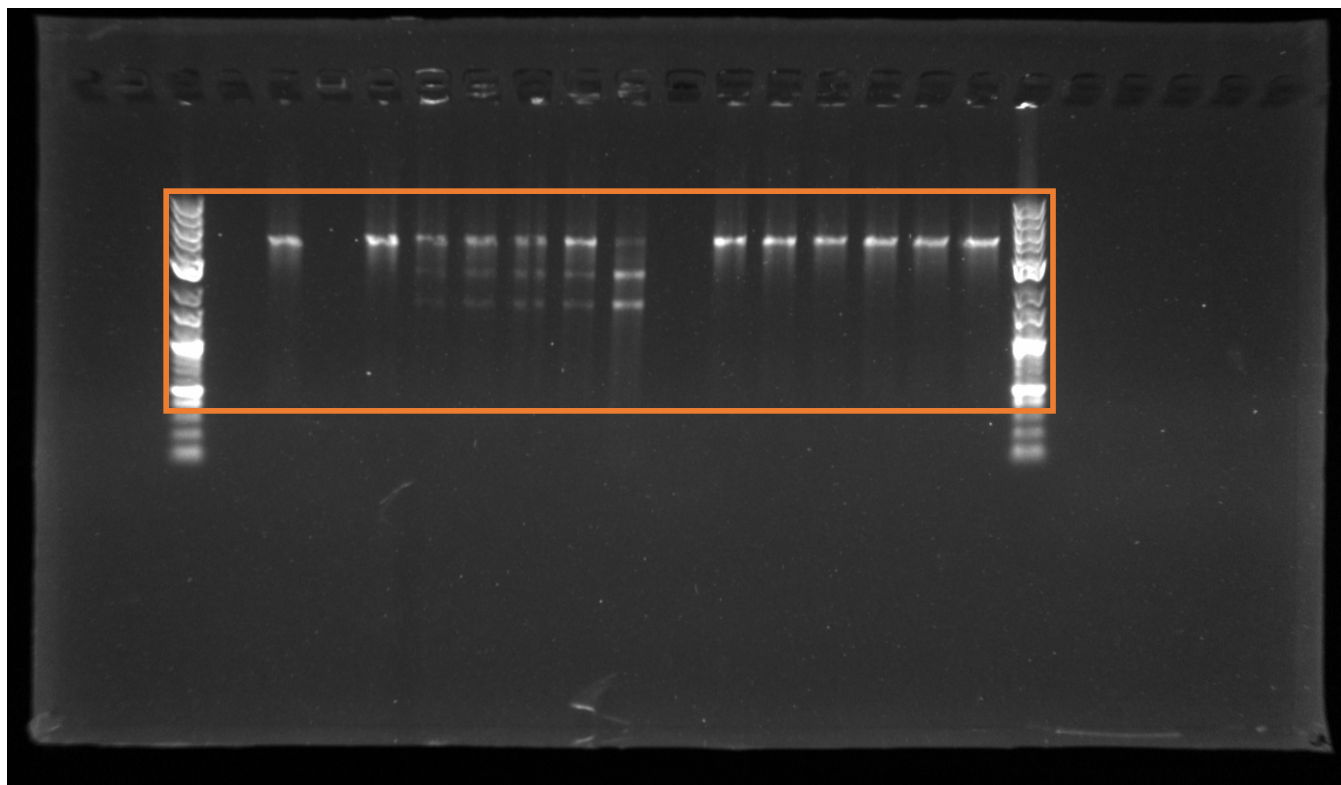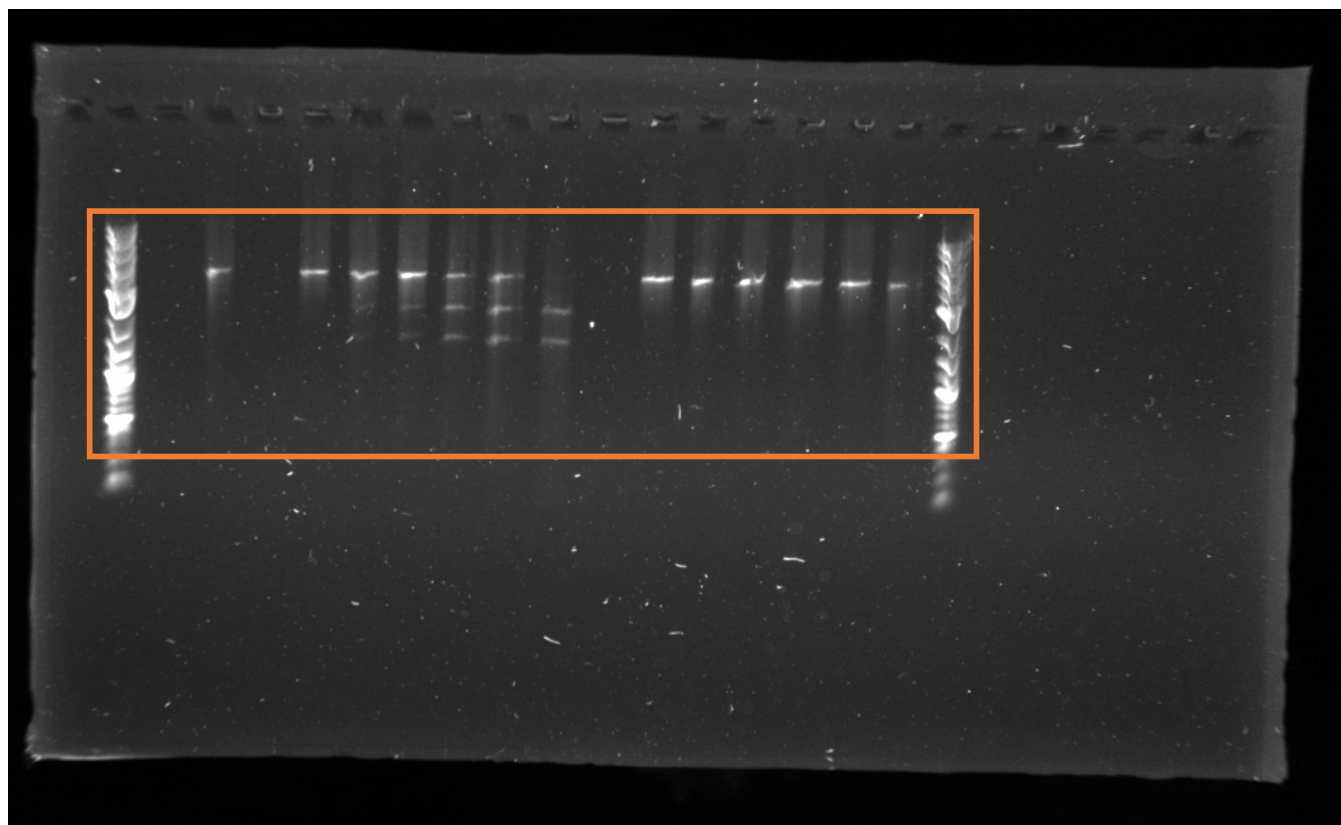

Supplement: Figure 5—figure supplement 1—source data 1. — The cropped images shown in the figure are indicated by the orange boxes. [file elife-68339-fig5-figsupp1-data1.pdf]

Figure 6 - source data 1

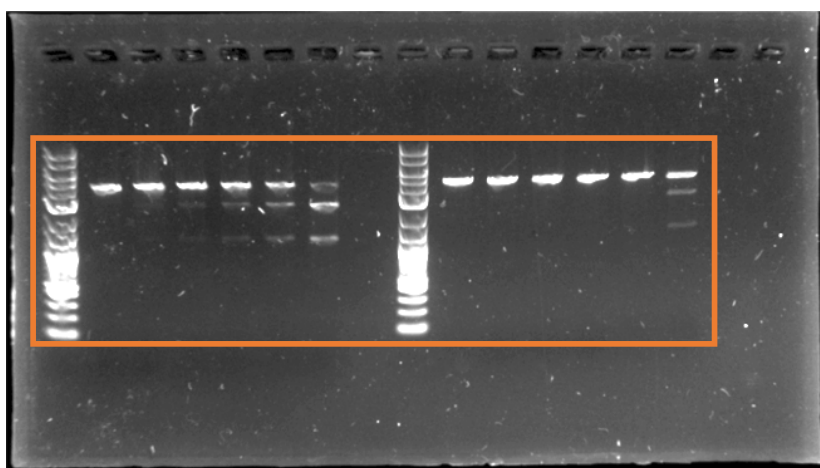

Supplement: Figure 6—source data 1. — The cropped image shown in the figure is indicated by the orange box. [file elife-68339-fig6-data1.pdf]

Figure 6 - Figure Supplement 1- source data 1

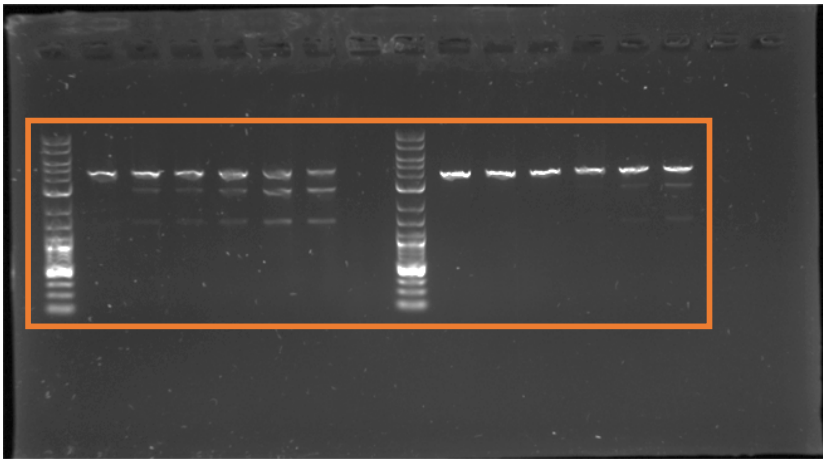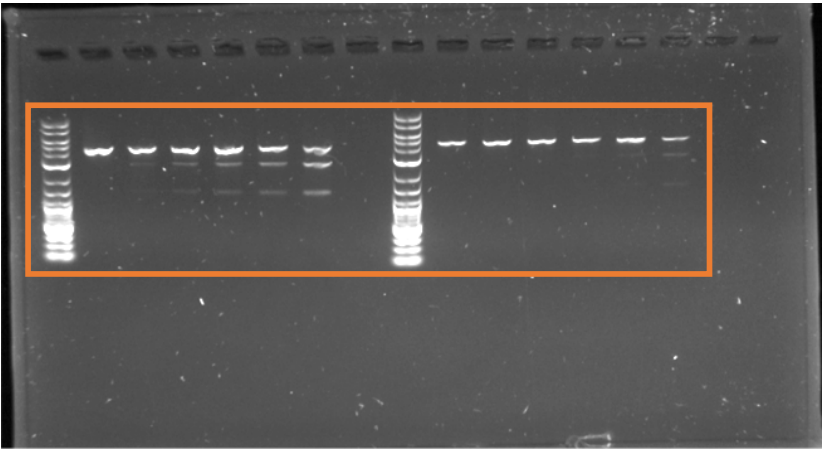

Supplement: Figure 6—figure supplement 1—source data 1. — The cropped images shown in the figure are indicated by the orange boxes. [file elife-68339-fig6-figsupp1-data1.pdf]
