# Supplementary material for: A chimeric nuclease substitutes a phage CRISPR-Cas system to provide sequence-specific immunity against subviral parasites
Source: eLife. 2021 Jul 7;10:e68339. doi: 10.7554/eLife.68339 (PMC8263062; doi:10.7554/eLife.68339)

Figure 4- figure supplement 2- source data 1

Figure 4- figure supplement 2A

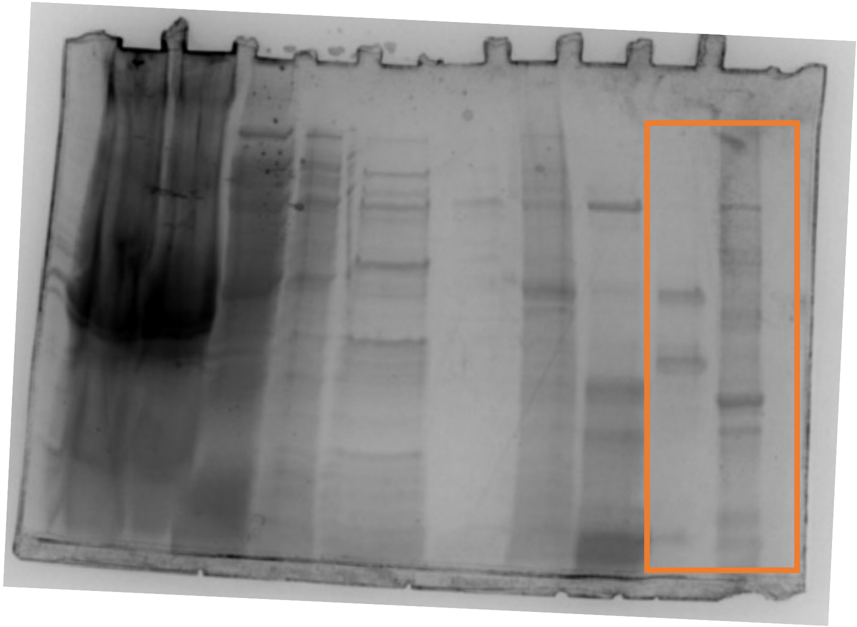

Figure 4- figure supplement 2B

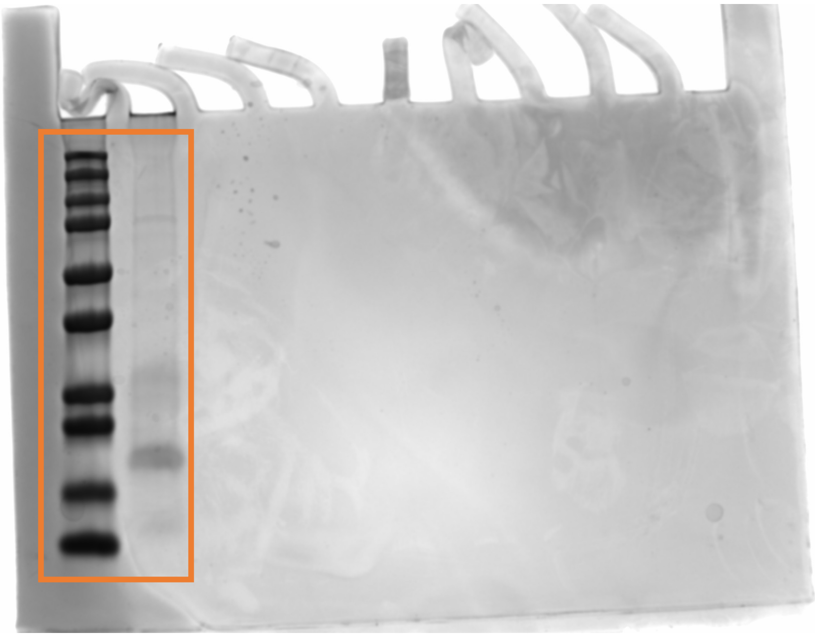

Figure 4- figure supplement 2C

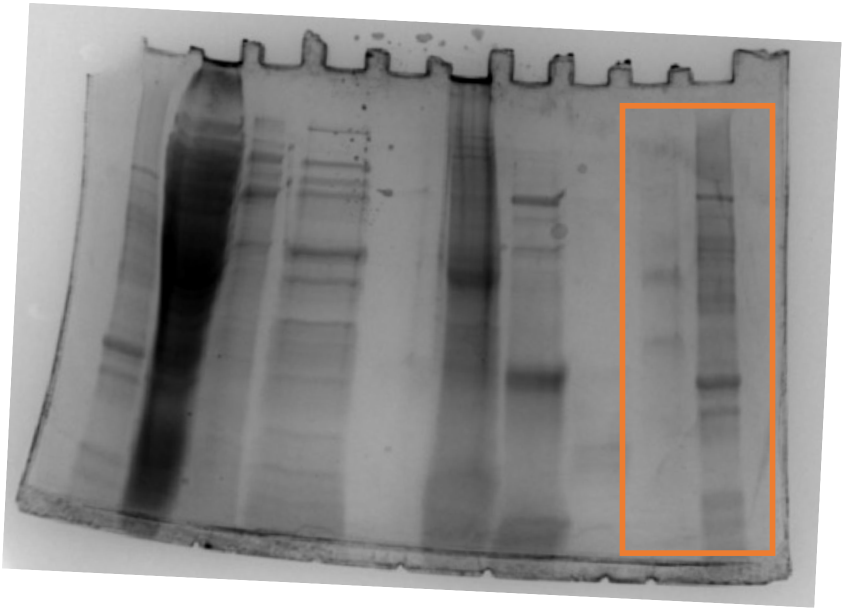

Supplement: Figure 4—figure supplement 2—source data 1. — The cropped images shown in the figure are indicated by the orange boxes. [file elife-68339-fig4-figsupp2-data1.pdf]
