# Supplementary material for: A chimeric nuclease substitutes a phage CRISPR-Cas system to provide sequence-specific immunity against subviral parasites
Source: eLife. 2021 Jul 7;10:e68339. doi: 10.7554/eLife.68339 (PMC8263062; doi:10.7554/eLife.68339)
Supplement: Transparent reporting form [file elife-68339-transrepform.pdf]

Westbrook Centre
Milton Road
Cambridge CB4 1YG, UK

P 01223 855340
W elifesciences.org
T @elife

eLife's transparent reporting form

We encourage authors to provide detailed information *within their submission* to facilitate the interpretation and replication of experiments. Authors can upload supporting documentation to indicate the use of appropriate reporting guidelines for health-related research (see EQUATOR Network), life science research (see the BioSharing Information Resource), or the ARRIVE guidelines for reporting work involving animal research. Where applicable, authors should refer to any relevant reporting standards documents in this form.

If you have any questions, please consult our Journal Policies and/or contact us: editorial@elifesciences.org.

Sample-size estimation

- You should state whether an appropriate sample size was computed when the study was being designed
- You should state the statistical method of sample size computation and any required assumptions
- If no explicit power analysis was used, you should describe how you decided what sample (replicate) size (number) to use

Please outline where this information can be found within the submission (e.g., sections or figure legends), or explain why this information doesn't apply to your submission:

> We did not perform experiments involving a heterogeneous population or pool of subjects, so the concept of sample size is not applicable to this manuscript.

Replicates

- You should report how often each experiment was performed
- You should include a definition of biological versus technical replication
- The data obtained should be provided and sufficient information should be provided to indicate the number of independent biological and/or technical replicates
- If you encountered any outliers, you should describe how these were handled
- Criteria for exclusion/inclusion of data should be clearly stated
- High-throughput sequence data should be uploaded before submission, with a private link for reviewers provided (these are available from both GEO and ArrayExpress)

Please outline where this information can be found within the submission (e.g., sections or figure legends), or explain why this information doesn't apply to your submission:

eLife Sciences Publications, Ltd is a limited liability non-profit non-stock corporation incorporated in the State of Delaware, USA, with company number 5030732, and is registered in the UK with company number FC030576 and branch number BR015634 at the address Westbrook Centre, Milton Road, Cambridge CB4 1YG.

Assays involving living cells (Figures 2, 3B, 3 figure supplement 2, 4B, 4C, and 5B, 6E) were performed in biological triplicate which we define as originating from a separately grown culture prior to back dilution for the assay. For phage spot assays this is noted in the figure legends and methods section. For DNA replication experiments (2B, 3B, and 3 figure supplement 2, 6D) this is noted in the methods section, and shown in the bar graphs where each dot represents a replicate. For biochemical nuclease assays (figures 4D, 4E, 4 figure supplement 3, 4 figure supplement 4, 5A, 5 figure supplement 1, 6C, and 6 figure supplement 1), experiments were performed in technical triplicate, which we define as independent incubations of protein with probe, using a separately thawed aliquot of protein. The assay performed in 4 figure supplement 5 was performed in technical duplicate.

eLife Sciences Publications, Ltd is a limited liability non-profit non-stock corporation incorporated in the State of Delaware, USA, with company number 5030732, and is registered in the UK with company number FC030576 and branch number BR015634 at the address Westbrook Centre, Milton Road, Cambridge CB4 1YG.

Statistical reporting

- Statistical analysis methods should be described and justified
- Raw data should be presented in figures whenever informative to do so (typically when N per group is less than 10)
- For each experiment, you should identify the statistical tests used, exact values of N, definitions of center, methods of multiple test correction, and dispersion and precision measures (e.g., mean, median, SD, SEM, confidence intervals; and, for the major substantive results, a measure of effect size (e.g., Pearson's r, Cohen's d)
- Report exact p-values wherever possible alongside the summary statistics and 95% confidence intervals. These should be reported for all key questions and not only when the p-value is less than 0.05.

Please outline where this information can be found within the submission (e.g., sections or figure legends), or explain why this information doesn't apply to your submission:

> We did not perform any statistical analysis beyond displaying the mean and SD of the replicates in our qPCR experiments. For numerical data, we only compare results with 100x or greater differences in magnitude, so further statistical analysis should be unnecessary

(For large datasets, or papers with a very large number of statistical tests, you may upload a single table file with tests, Ns, etc., with reference to sections in the manuscript.)

Group allocation

- Indicate how samples were allocated into experimental groups (in the case of clinical studies, please specify allocation to treatment method); if randomization was used, please also state if restricted randomization was applied
- Indicate if masking was used during group allocation, data collection and/or data analysis

Please outline where this information can be found within the submission (e.g., sections or figure legends), or explain why this information doesn't apply to your submission:

> Our experimental design did not involve groups. Comparisons were made between strains of different genetic backgrounds, so there wasn't another criteria to group things by.

Additional data files ("source data")

- We encourage you to upload relevant additional data files, such as numerical data that are represented as a graph in a figure, or as a summary table
- Where provided, these should be in the most useful format, and they can be uploaded as "Source data" files linked to a main figure or table
- Include model definition files including the full list of parameters used
- Include code used for data analysis (e.g., R, MatLab)
- Avoid stating that data files are "available upon request"

Please indicate the figures or tables for which source data files have been provided:

> We have uploaded source data for Figures 2B, 3B, 3-supplement 2, 4, 4-supplement 2, 4-supplement 3, 4-supplement 4, 4-supplement 5, 5A, 5-supplement 1, 6C, 6D and 6-supplement 1

eLife Sciences Publications, Ltd is a limited liability non-profit non-stock corporation incorporated in the State of Delaware, USA, with company number 5030732, and is registered in the UK with company number FC030576 and branch number BR015634 at the address Westbrook Centre, Milton Road, Cambridge CB4 1YG.
